# Surgery and Soft-Tissue Tumours

**DOI:** 10.1080/135771402753667664

**Published:** 2002-05


### SURGERY AND SOFT-TISSUE TUMOURS

### ORAL PRESENTATIONS

### C79 Reliability of Risk Factors and Scoring Systems in Predicting Fracturing in Metastatic Femoral Bone Lesions

Y.M. van der Linden, H.M. Kroon, P.D.S. Dijkstra, E.M. Noordijk
*Leiden University Medical Center, Leiden, The Netherlands*

*Objective:* In randomised Dutch Bone Metastasis Study on the palliative effect of single versus multiple fraction irradiation on painful bone metastases all impending fractures were excluded from participation. During intensive follow-up 35 fractures occurred in 1157 patients. A subset of 102 patients with a femoral lesion, in which 8 fractures occurred within 12 weeks after irradiation (8%), was used to assess the pre-treatment fracture risk.
*Methods:* Pre-treatment radiographs were collected. A radiologist, orthopaedic surgeon and radiation oncologist independently and without knowledge of the outcome after treatment (fracture yes/no) scored size and extent in mm, radiographic appearance/structure/aspect, localisation, percentage of cortical involvement and estimated risk of fracturing. Known risk factors (size >2.5 cm, cortical involvement >50% or continuing pain after irradiation) and an adjusted Mirels' scoring system* were applied to evaluate effectiveness (table1).
*Results:* If a score of 9 points as Mirels suggested was applied sensitivity for predicting a fracture was high but specificity very low (table2). The positive predictive value of Mirels was low (<10%). Size >2.5 cm, cortical involvement >50% or continuing pain were not predictive of fracturing.
*Conclusion:* In this study reliability of accepted risk factors and scoring systems in predicting fracturing in patients with a femoral bone metastasis is low. The use of Mirels' scoring system will lead to excessive surgical overtreatment (positive predictive value <10%). Also size >2.5 cm, cortical involvement >50% or continuing pain after irradiation are not specific enough. Development of a more sensitive scoring system is ongoing.

### C80 Predictors of Wound Complications Following Free and Pedicled Soft Tissue Flaps for Reconstruction After Excision of Soft Tissue Tumours

A. Abudu[1], R.S. Bell[2], A.M. Griffin[2], B. O'Sullivan[2], C.N. Catton[2], A.M. Davis[2], J.S. Wunder[2]
*[1]Royal Orthopaedic Hospital, Birmingham, United Kingdom, [2]Mount Sinai Hospital, Toronto, Canada*

113 consecutive patients with soft tissue sarcoma treated by excision and reconstructive flaps were studied to assess the risk of complications and to compare local tumour control with those in whom primary wound closure was possible. Minimum follow-up was 24 months and mean age was 55 years (16–95). The sarcoma was located in the lower extremity in 83 and upper extremity 30 patients. Significant wound complications developed in 37 patients (33%). The most common complications were wound infections or partial necrosis occurring in 16% (18/113) and 13% (15/113) respectively. Complete flap necrosis requiring flap removal occurred in 6 patients (5%). Three patients (2.3%) required amputation as a result of complications. Significant risk factors for development of wound complications include location of tumour in the lower limb compared to upper limb (relative risk 2.3, p=0.02) and use of pre-operative radiotherapy compared to no or post-operative radiotherapy (relative risk 2.05, p=0.02). There was no difference in rates of complications in patients with free or pedicled flaps, tumours < or >5 cm, distal or proximal location of tumour. The rates of negative excision margins (80%) and wound complications in patients who required reconstructive flaps were not different from that for the other patients treated at our centre who did not require reconstructive flaps.
*Conclusion:* The use of soft tissue reconstructive flaps did not reduce the risk of positive excision margins or the rates of wound complications. The risk of amputation secondary to flap complication or failure is low.

### C81 Silver-Coated Tumour Endoprostheses for Prevention of Deep Infection

G. Gosheger[1], H. Ahrens[2], A. Streitbuerger[1], W. Winkelmann[1]
*[1]University Hospital, Muenster, Germany, [2]University Children's Hospital, Muenster, Germany*

Deep infection of a megaprosthesis remains a serious complication (Lit.: 8% to 35% infection rate). Silver-coated catheters proved their effectiveness in reducing infections. This study should evaluate the effectiveness of a silver-coated endoprosthesis and should exclude side effects of a large surface. 40 rabbits (titanium- versus silver-coated endoprostheses) were infected with 5×104 colony forming units of a staphylococcus aureus. The silver group showed significant (p<0,05, chi-square-test) lower infection rates (7% versus 47%) in comparison to the titanium group. Furthermore the measurements of the c-reactive-protein, neutrophilic leucocytes, temperature and body weight showed significant (p<0,01) lower signs of inflammation in the silver group. The analysis of the silver concentration in the blood (median 1, 883 ppb) and in the organs (0, 798–86, 002 ppb) showed elevated silver concentrations in all compartments. But there were no pathologic changes in the laboratory parameters (e.g. creatine kinase, GOT, GPT). In addition there were no pathologic changes in the macroscopic or the microscopic sections of the implant and the organs. This study could confirm the described effect of silver-coated medical devices. The blood concentration of silver does not state a critical concentration according to literature. Furthermore the overall amount of silver in case of a coated total femur endoprosthesis is 1,01 g silver and does not state a critical amount according to literature.
*Conclusion:* the new silver-coated Mutars-tumour endoprosthesis is very promising and should be clinically evaluated.

### C82 Our Experience with the Capanna Method of Limb Reconstruction Using Free Fibular Grafts and Cadaveric Allograft Report of 16 Consecutive Patients

J. Bickels[1], Y. Kollender[1], I. Meller[1], A. Amir[2], D. Leshem[2], T. Shpitzer[2], A. Zaretski[2], A. Ad-El[2], Y. Barnea[2], R. Shafir[2], J. Weiss[2], E. Gur[3]
*[1]Nat. Unit of Orthopedic Oncology, Tel Aviv Sourasky Medical Center, Tel Aviv, Israel, [2]Microsurgery Unit, Tel Aviv Sourasky Medical Center, Tel Aviv, Israel, [3]Gulhane Military Medical Academy, Ankara, Turkey*

*Introduction:* Fibular grafts have become a common mode of reconstruction after resection of long bone tumours and especially those that require intercalary resection. The current study summarizes the authors experience with 16 consecutive patients who underwent limb-sparing resection of a long bone tumour and reconstruction with a fibular graft.
*Methods:* Between 1997 and 2001, 16 patients underwent reconstruction of a bone defect with a fibular graft using the Capanna technique of combined fibular graft, allograft, and hardware fixation. All patients were initially diagnosed with a primary bone sarcoma. Twelve patients who were diagnosed with Ewing's sarcoma had a delayed reconstruction (3–5 years after primary reconstruction with a spacer) and the remaining 4 patients with osteosarcoma had a primary reconstruction at the time of tumour resection. Anatomic locations of reconstructions were: femur – 12, tibia – 4. Postoperatively, the operated extremities were immobilized in a cast for 6 to 9 months during which a gradual weight bearing was practiced. Flap viability was monitored using an implantable doppler device, a bone scintigraphy that was performed at the 10th postoperative day, and plain radiographs.
*Results:* All 16 fibular grafts were viable and united. Plain radiographs performed at 3 to 5 months after surgery showed bridging callus formation around all fibular grafts. None of the study patients had a major recipient or donor site complication. In two patients a failure of the hardware fixation system was observed and surgically repaired.
*Conclusion:* The Capanna method of fibular graft reconstruction provides a biological reconstruction of large bone defects. It is a safe and reliable technique that is associated with good functional outcome in the majority of the patients.

**C83 Composite Grafts in the Treatment of Osteosarcoma of the Proximal Humerus**

M. Salai, I. Dudkiewicz, A. Oran, M. Prtisch
*Chaim Sheba Medical Center, Tel Hashomer, Israel*

Treatment of osteosarcoma of the proximal humerus poses many difficulties and challenges to the treating team. Between 1993 and 2000 we had treated 11 patients who suffered from osteosarcoma of the proximal humerus by composite of massive allografts and long humeral prosthesis. Three patients were female and 8 men, with age range of 17–74 years. Ten patients were at stage 2-b at presentation, one patient was 3-b. one of the patients had pathologic fracture at presentation. All patients received pre-operative chemotherapy followed by limb salvage surgery and postoperative chemotherapy. Margins were wide in all patients. Postoperative complication included non-union at the allograft/host junction (which united after auto grafting) and superficial wound infection, which resolved after antibiotic therapy. A team of orthopedic oncologist and shoulder surgeon performed all operations. At latest follow-up (December 2001), all patients except one (with 3-b at presentation) are alive, with good functioning upper limb.
*Conclusion:* the team approach of orthopedic oncologist and shoulder surgeon contributed largely to the good surgical results, and hence the survival and function. We suggest implementation of such team approach also in other fields of orthopedic oncology: spine, hip, knee etc.

### POSTER PRESENTATIONS

**C84 CAD Supported Planning with Stereo Lithography Model in Bone Tumours of the Pelvis**

C. Gebert, J. Hardes, B. Leidinger, G. Gosheger, W. Winkelmann
*Dept. of Orthopaedics, University Hospital, Muenster, Germany*

Long term survival of malignant bone tumours is strictly correlated to the wide resection of the tumour. Especially, in tumours of the pelvis, a correct estimation of the resection is necessary. Typically, there were used CT scans or MR images and sometimes surface-models manufactured in CNC technique. Despite this 3-D-reconstruction technique, it was not possible to get information about the intra-cortical tumour expansion. In lithography, each partial volume of the CT-scan is expressed in a proportional volume of the plastic model. With Stereolithography the estimation of the tumour expansion is much easier, because the intracortical tumour defect component is visualised by a 'defect' in the model. Using the model, it is possible to produce individual metal blocks supporting the correct resection. It is essential to perform an exact resection for the primary fit of pelvic (partial-) prosthesis. In our department we treated three patients by internal hemipelvectomy with the help of stereolithography models. The primary bone contact of the prostheses was quite good in all patients with a shortening of the time needed for the operation compared to former resection techniques in pelvis tumours.
*Conclusion:* With the help of CAD-techniques in combination with models manufactured in lithography technique there can be achieved an exact resection if there is a good interaction between the surgeon and the prosthesis manufacture.

**C85 Transosseus Osteosynthesis (TO) in the Treatment of Pathological Fractures (PF).**

V.V. Tepliakov, I.M. Lebedenco, E.E. Kovalevskii, M.D. Aliev
*Blokhin Cancer Center, Moscow, Russian Federation*

*Purpose:* to show an opportunity of TO in combined treatment of patients with PF of long tubular bones.
*Methods:* there were 31 PTAs: 10 men, 21 women. Age range 17–70. PF occurred in 13 cases of breast cancer metastases, 3- of renal cancer, 2- of cancer of the stomach and the oesophagus, 2- metastases without detected primary, 2-metatsases of Eking sarcoma, 3- of lymphosarcoma, 2- of osteosarcoma. And metastases from MFH, melanoma, fibro-osseous dysphasia, lymphogranulomatosis – each for 1 case. The localization of the PF was: neck of femur- 10 cases, proximal-8 and distal-4 metadiaphyses of femur, 5- diaphysis of femur. The humerus: 3 in the diaphysis, 1- neck of humerus. TO was held in time of 11 days after PF. 19 patients received radiation therapy single dose 4 Gy, SD 20–59 Gy. 2 patients received radiation therapy in combination with intraarterial chemotherapy, 13- in combination with i/v chemotherapy and hormonal therapy. 6 patients received only chemotherapy, hormonal therapy or immunotherapy. 5 patients didn't receive any additional treatment.
*Results:* 16 patients reached complete consolidation after 152 days. 4 patients after TO and conservative therapy received wide resections with allografts or autografts with vascularised bone. The mean follow up period was 36.5 months. Good functional results were achieved in 14 patients. Satisfactory results were achieved in 6 patients. 11 patients died from disease progression. In the time of 6.5 months after TO.
*Conclusion:* TO allows one to reach consolidation of the fracture and to add chemotherapy or radiation therapy. In the cases of favourable prognosis and the absence of consolidation its possible to make salvage operations (arthroplasty, auto- or allo- bone grafts implanting).

**C86 The Role of Microsurgery in Orthopedic Oncology: A 3-Years Experience with 39 Cases of Limb-Sparing Surgery**

J. Bickels[1], Y. Kollender[1], I. Meller[1], A. Amir[2], D. Leshem[2], T. Shpitzer[2], A. Zaretski[2], A. Ad-El[2], Y. Barnea[2], R. Shafir[2], J. Weiss[2], E. Gur[3]
[1]*Nat. Unit of Orthopedic Oncology, Tel Aviv Sourasky Medical Center, Tel Aviv, Israel,* [2]*Microsurgery Unit, Tel Aviv Sourasky Medical Center, Tel Aviv, Israel,* [3]*Gulhane Military Medical Academy, Ankara, Turkey*

*Introduction:* Limb sparing-resections of large tumours are associated with the creation of large bone and soft-tissue defects. Availability of microsurgical techniques allows reconstruction of such defects that otherwise would have require an amputation. The current study summarizes a 3-years experience with microsurgical reconstructions following limb-sparing resections.
*Material and Methods:* Between 1998 and 2001, 39 patients required microsurgical intervention. There were 25 males and 14 females who ranged in age from 8 to 42 years. Diagnoses included: primary bone sarcomas – 31, soft tissue sarcomas – 8. Anatomical locations were: femur – 25, tibia – 4, radius – 4, humerus – 1, fibula – 1, heel – 3, and forearm – 1. Primary microsurgical reconstruction following tumour resection was performed in 11 patients and delayed reconstruction (after primary bone reconstruction with a spacer or as a treatment of late infection) in the remaining 28. A fibular graft was used for reconstruction of intercalary bone defect in 19 patients, knee arthrodesis – 2, distal radius reconstruction – 4, and proximal humerus reconstruction – 1. Eight patients had soft tissue reconstructions (Latissimus dorsi flap – 1; scapular flap – 1; gracilis muscle flap – 1).
*Results:* At the most recent follow-up, all bone and soft-tissue flaps were united and viable, respectively. None of the study patients had infection or local tumour recurrence. Only one patient had a mechanical failure of internal fixation implant that required a secondary reinforcement.
*Conclusion:* Microsurgical techniques of reconstruction allow a limb-sparing resection of large bone and soft-tissue tumours that otherwise would necessitate and amputation. Microvascular reconstructions are associated with high rate of success and provide a functional extremity in the majority of the patients.

### C87 Limb-Salvage Surgery for Malignant Musculoskeletal Tumours in Children

A. Shchurovsky, I. Lukavetskyj, O. Mohylyak, T. Pidlisetsky
*Lviv Regional Specialised Children's Clinic, Lviv, Ukraine*

*Objective of study:* Limb-salvage surgery for malignant bone tumours has become the standard method of treatment. The longevity, complications and functional results of these methods vary according to anatomic location, age and type of surgery.
*Methods:* Between January 1993 and January 2002 were treated 63 patients. Due to the fact that the majority of patients were admitted in late stage of disease only in 30 of them ( 21 males and 9 females) underwent the limb-salvage procedures. The age ranged from 1 till 18 years (mean- 14,3 years). The diagnosis were: Osteosarcoma – 13 patients , Ewing's Sarcoma – 6, Malignant Giant Cell Tumours – 3, Soft-Tissue Tumours – 3, Chondrosarcoma – 2 and Malignant Fibrous Histiocytoma – 1 patient. The tumour locations were: proximal femur – 1 patient, distal femur – 11, proximal tibia – 2, proximal fibula – 4, distal fibula – 1, proximal humerus – 5, pelvis – 1, ribs – 3, forearm – 1 patient and hand in one case. For these patients were performed different types of limb-salvage surgery: prosthetic replacement – 12 patients, resection – 12 patients, resection with allo-, auto- or ceramoplasty in 5 patients, and rotationplasty in one case.
*Results:* The earliest follow-up for prosthetic replacement was 60 months , for resection-reconstruction – 36 months and after resection – 64 months. The function was excellent and good in 11 patients after prosthetic replacement (91,6%), in 4 patients after resection – reconstruction (80%) and in 10 patients after resection (83,3%).
*Conclusion:* The best functional results were received in patients after prosthetic replacement. This kind of treatment has faster and better aesthetic effect in comparison with the other limb-salvage surgery techniques. This positively effects psychological and social aspects of such patients.

### C88 Our Experience of Using Revascularised Flaps in Oncology

V.A. Sobolevskyi, M.A. Kropotov, E.G. Matiakin, M.D. Aliev
*Blokhin Cancer Center, Moscow, Russian Federation*

The use of microsurgical reconstructive technique in oncology affords to reach more radical incision of the tumour from one side, and to increase functional and cosmetical results from another. In our study we reviewed 29 pts. The localization of the lesion was: 15- lower extremities (in 4 cases- benign tumours), 5pts- upper extremities, 8- head and neck,1 pt – abdominal wall localization. There were primary bone tumours in 14 cases, 7 cases- skin tumours, 5-soft tissue sarcomas, 3 pts with the mucous tumours of oral cavity. There were locally advanced lesions in all cases, that why it was impossible to execute salvage operation or to close the defect with local tissues. We used bone plasty in 15 cases: 8 – tibial bone, 4- bones of the forearm, 3 mandibular bone. In one case for closing defect of head skin and temporal bone we used the fragment of omentum. Most often we used vascularised fibular bone- 13 cases, 1 combined scapular flap, 1 iliac bone flap. Soft tissue defects were replacing in 14 pts, in three cases radial forearm flap and thoracodorsal flap (8 pts), scapular flap (2 pts). Complications were observed in 6 (20,7%) pts: flap necrosis -4 pts (13.8%), wound infection in 2 pts. Follow up period was 2 years, and there were 2 cases of recurrence. 1 pt died from generalization.
*Conclusion:* The use of microsurgical reconstruction technique allows to reach good functional and cosmetic results with favourable oncological prognosis.

### C89 Surgical Management of Humeral Metastases Principles of Surgical Intervention, Functional, and Oncological Outcomes

J. Bickels[1], J.C. Wittig[2], R. Oren[1], Y. Kollender[1], K. Kellar-Graney[2], M.M. Malawer[2]
[1]*Tel Aviv Sourasky Medical Center, Tel Aviv, Israel*
[2]*Washington Cancer Institute, Washington, USA*

*Introduction:* Metastatic-bone-disease of the humerus may be associated with disabling pain and loss of function. Surgery must provide good local tumour-control, immediate mechanical-stability, and a short rehabilitation-period. Between 1980 and 2000, the authors operated 47 patients with metastatic disease of the humerus. The current study summarizes the principles of the surgical technique and functional and oncological outcomes.
*Methods:* There were 26 females and 21 males who ranged in age from 34 to 76 years. Forty-one patients presented with an impending or pathological fracture and 6 had intractable pain. Anatomic locations of humeral metastases included: around the proximal humeral metaphysis and head (Type I) – 14, humeral diaphysis (Type II) – 32, and humeral condyles (Type III) – 1. Types I and III metastases were treated with resection and endoprosthetic reconstruction. Type II metastases were treated with intralesional tumour removal and cemented internal fixation. Motion of the operated extremity was allowed immediately after surgery. Postoperatively, 32 patients were treated with radiation therapy and 10 with chemotherapy. Follow-up of the study patients included physical examination, radiological evaluation and functional evaluation according to the American Musculoskeletal Tumour Society system.
*Results:* Following surgery, all patients had a stable and painless extremity. Function was good-to-excellent in 38 patients and moderate in 9 patients. Thirty-one (66%) patients survived more than 1 year and 19 (40%) survived 2 years after surgery with local tumour recurrence occurring in only one patient.
*Conclusion:* The anatomic location of humeral metastasis dictates the mode of tumour resection and reconstruction: periarticular tumours are treated with endoprosthetic replacement and diaphyseal tumours with curettage and cemented internal fixation. An aggressive surgical approach and judgmental use of adjuvant treatment modalities achieve good functional outcomes and local tumour control in the majority of these patients.

**C90 Limb Salvage Surgery and Implantation of Mega – Endoprothesis in 62 Patients with Bone Tumours**

D. Orlic, M. Smerdelj, R. Kolundzic, O. Ivna
*Dept. of Orthopaedic Surgery, University of Zagreb School of Medicine, Zagreb, Croatia*

Special modular uncemented endoprostheses from 1987 to 2001 were implanted in 62 patients after 'en bloc' resection. The most often indications were malignant bone tumours in 44 (69,3%) patients, benign bone tumours in 11 (17,7%) massive destruction of the proximal femur or instability of hip endoprosthesis in 4 (6,4%) and tumours like lesions in 3 (4,8%). Osteosarcoma was mostly resected malignant bone tumour in 21–33,9% of all operated (!) patients, Ewing sarcoma in 9, chondrosarcoma in 6, metastasis in 2, fibrosarcoma, synoviosarcoma, angiosarcoma, periosteal osteosarcoma, MFH, angiosarcoma and leiomyosarcoma in 1 patient respectively. Salvage surgery was performed in 38 knee and 24 hip joints. The median length of proximal femoral resection was 17 cm; of distal femur 15,4 cm, and 13 cm of proximal tibia.
*Results of treatments were analysed:* 42 patients (67,7%) showed no evidence of disease at the last follow-up; 2 (3,2%) changed the state of living and we are without any information, and 18 patients (29%) had died. Additional postinfective complications (as contracture of the knee joint in two patients, fused knee after salvage surgery, luxation of endoprosthesis and the girl with hepatitis and salvage hip surgery is waiting for reoperation) were registered in 4/62 patients. With the aid of contemporary chemotherapy limb salvage surgery is and will be a great challenge for orthopaedic oncologists of the 21st century.

**C91 'Endlock' Stem Design for Anchorage of Massive Reconstruction Endoprostheses.**

G.U. Exner[1], HAC Jacob[1], R. Bedzinski[2]
[1]*Univ. of Zurich, Zurich, Switzerland,* [2]*Technical University, Wrozlaw, Poland*

*Rationale:* Most anchoring stems for cementless fixation produced currently are designed for bone adherence or ingrowth over the full length. While ensuring good primary stability, they have the drawback of stress-shielding resulting in resorption of bone in the proximity of the insertion site at the resected end of the bone.
*Methods:* We have therefore designed a stem system made of cobalt chromium exhibiting a smooth polished surface at the far end and a fixation region presenting a scalloped surface plasma coated with pure titanium for over a length of about 3 cm towards the joint. This limits force transmission from the endoprosthesis to the host bone only within a short distance at the insertion site. Finite element analysis has proved that the system can bear the loads imposed and that the bone is adequately loaded along its whole length, thereby ensuring that the bone is not subjected to stress-shielding and subsequent atrophy.
*Patients:* The system was used in 6 osteosarcoma patients for primary implantation, and in 4 patients for the revision of loosened conventional stems.
*Results:* In 2 cases of primary implantation loosening occurred needing replacement, for which larger stems of the same design were used; in all cases loosening was related to splitting of the bone at the time of surgery. All other cases including all the revision cases remained stable at 2–3 years f/u with no signs of bone atrophy due to stress shielding.
*Conclusion:* The hypothetical assumptions related to stem design have been confirmed through finite element analysis. In clinical application some technical aspects of preparing the bone need improvement. If primary stability is achieved, long term prognosis appears to be improved by the system. Stress-shielding is reduced compared to conventional long stems.

**C92 Surgery and Radiotherapy as Combined Treatment for the Primary Management of Pigmented Villonodular Synovitis of the Foot and Ankle**

M Lee, S Mahroof
*TWR Briggs Bone Tumour Unit Royal National Orthopaedic Hospital, Stanmore, UK*

Surgery is the mainstay of treatment for pigmented villonodular synovitis (PVNS), whether local excision for the nodular form, or total synovectomy for diffuse cases. Radiotherapy has been used in the management of recurrences, but is not routinely part of primary treatment. A previous study combining radiotherapy and synovectomy has reported encouraging results with regard to the knee. We present the results of combined synovectomy and low dose external beam radiotherapy in the primary management of PVNS of the foot & ankle. 7 patients with clinical, MRI and histologically confirmed PVNS of the foot & ankle were followed in a prospective study between 1997 and 1999 at the Royal National Orthopaedic Hospital. The patients underwent synovectomy, followed by radiotherapy with a total dose of 30Gy. One surgeon performed the operation. At an average follow-up of 26 months, 6 of the 7 patients (85%) reported persistent swelling and pain, and was found to have a recurrence of the lesion. we believe that using a low dose of radiotherapy in conjunction with surgical excision of a primary PVNS lesion in the foot and ankle may reduce the risk of recurrence without impairing joint mobility.

**C93 Repair of Bone Defects, Which Occurred During Treatment of Bone Tumours, with Ilizarov Bone Transportation Method**

K. Erler, M. Basbozkurt, C. Yildiz, E. Gur
*Gulhane Military Medical Academy, Ankara, Turkey*

*Objective of Study:* To repair bone defects, which occurred after en block resection during malign or aggressive benign bone tumour surgery, with bone transportation method described by Ilizarov.
*Methods:* 8 cases, on whom en block resection was performed due to bone tumours were treated by Ilizarov bone transportation method between October 1991 – June 2001 in our clinic. Six were male, and 2 were female. There was one case with distal femur osteosarcoma, one with tibial osteofibrous dysplasia, one with tibial aggressive osteoblostoma, one with giant cell tumour at tibia, one with tibial diaphyseal osteosarcoma, and two with Ewing sarcoma at femur. After en block resection was performed, bifocal CEF (circular external fixator) was applied and corticotomy was done at either proximal or distal metaphysis. Distraction was started on the 4th – 7th day and continued 4×0.25 mm every day. Ilizarov frame was removed when sufficient bone consolidation was observed in follow-up radiograms. During the follow-up period, the patients continued rehabilitation program.
*Results:* The average follow-up period was 46 months, and the average duration of CEF was 15 months. The average length of bone defect after tumour resection was 16.5 cm (11–23). Skin fold occurred in one patient and was treated by an operation. Bone marrow injection was used in one case with delayed union. One case had supracondylar femur fracture due to falling down during the follow-up period so open reduction and internal fixation was performed. Pin tract infection was observed in five patients and they were treated by antibiotic therapy and dressing. Restriction of the range of motion was observed in one case.
*Conclusion:* We observed that the bone transportation method of Ilizarov is an alternative method for the repair of bone defects that occurred during the treatment of bone tumours.

**C94 Endoprosthetic Reconstruction of the Upper Extremity Using the Trevira Tube**

G. Gosheger[1], C. Gebert[2], J. Hardes[1], W. Winkelmann[1]
*[1]University Hospital, Muenster, Germany, [2]University of Muenster, Muenster, Germany*

To avoid dislocation of an unconstrained endoprosthesis a special trevira tube (polyethylene terephthalate) was developed. The trevira tube is a knitted tube belonging to the Modular Tumour and Revision System (Mutars®, Implantcast Corp, Buxtehude, Germany). It is characterized by a porous structure of 200 mm and a tensile strength of 4000 N. This tube is attached to the rest of the capsular structures. The tube is fixed to the megaprosthesis and the muscles are reattached to the prosthesis. A non-absorbable suture is used for all attachments to the trevira tube. In the case of proximal humerus replacement (16 patients), the trevira tube allows for reconstruction of the capsule and refixation of the muscles. By using the trevira tube in proximal humerus replacement, the complete rotator cuff was reattached in 11 patients, only the subscapular muscle was reattached in three patients, and only the supraspinatus muscle could be reattached in two patients. No dislocation was observed in patients with proximal humerus endoprosthesis. The mean active abductor motion against gravity was 37.5° (range, 0°–75°), the anteversion was on average 35.0° (range, 5°–79°), and active ROM of internal rotation was on average 15.2° (range, 10°–35°) and external rotation was on average 25.2° (range, 15°–45°), but there was an increased range of passive motion in these patients especially in external rotation (up to 120°). The mean overall functional outcome of patients with a proximal humerus replacement was 70.4% (range, 46%–83%) according to the Musculoskeletal Tumour Society score. There was no significant increase in the rate of infection (p = 0.46; chi square test). The histopathologic findings showed a tissue ingrowth into the tube. In proximal humerus replacement the trevira tube provides safe protection against dislocation, the abductor capability could not be improved as predicted previously.

**C95 Gait and Function in Patients with a Femoral Endoprosthesis After Tumour-Resection 18 patients evaluated 11,8 years after surgery**

J.C. Rompen, S.J. Ham, J.P.K. Halbertsma, J.R. Van Horn
*University Hospital Groningen, Groningen, The Netherlands*

We performed gait analysis in 18 patients with a femoral endoprosthesis; 12 distal, 3 proximal and 3 total femoral endoprostheses. Follow-up after tumour resection and reconstruction was mean 12 (0.5–19) years. Measured gait parameters were walking velocity, steplength, duration of stancephase and swingphase. Furthermore, goniometry of the hip, knee and ankle in both legs was performed during free paced walking. The functional outcome score of the Musculoskeletal Tumour Society (MSTS) and the Ambulation score were also performed in all patients. The mean free paced walking velocity was 88% of normal. The steplength of the non-involved leg was longer than that of the involved leg. The swingphase of the involved leg was longer compared to the non-involved leg, and the stancephase of the involved leg was shorter compared to the non-involved leg. Goniometry revealed three abnormal patterns in the involved leg; a stiff knee gait was observed in 10 patients, and a flexed knee gait in 6. An abnormal flexion-extension pattern in the hip was observed in 9 patients. Goniometry of the non-involved leg was normal. The mean MSTS score was 22 points (72%). The MSTS score was significantly positively correlated to the Ambulation score, but was not significantly correlated to any of the measured temporal variables. Our findings indicate that time of load on the involved leg, whether consciously or not, is reduced. Follow-up studies are necessary to evaluate the effects of the observed asymmetrical gait pattern and the abnormal goniometric results on the development of endoprosthesis-related complications.

**C96 Should Soft Tissue Sarcomas be Treated at a Specialist Centre?**

AA Bhangu[1], RJ Grimer[2]
*[1]University of Birmingham, Birmingham, United Kingdom, [2]Royal Orthopaedic Hospital, Birmingham, United Kingdom*

*Aim:* 'Cancer should be treated by cancer specialists' is often stated, but little proof exists that outcomes are different. We have investigated whether there is evidence that patients with soft tissue sarcomas do better if treated in a specialist centre compared with district general hospitals.
*Methods:* We analysed the outcomes for all patients with soft tissue sarcomas in one health region of the UK over a 3 year period, with minimum follow up of 4 years. During this time half of patients were treated at a specialist musculoskeletal oncology centre whilst the remainder had treatment centred in a DGH. We have investigated appropriateness of treatment, adequacy of surgery, use of adjuvant treatment and outcomes in terms of local control and overall survival. Data was obtained from the Cancer Intelligence Unit and the specialist centre. Results are stratified for known risk factors for local control and survival (margins, grade/size for LR; depth, size and grade for survival).
*Results:* 296 patients were diagnosed as having STS over the 3 year period (incidence = 1.71 per 100,000 per year). Half of patients had the majority of treatment at the specialist centre under the care of 2 surgeons, whilst the other half were treated by a total of 105 different surgeons. Care at the specialist centre involved staging, biopsy, excision (wide margins if possible) and adjuvant radiotherapy. Care at DGHs was haphazard and only a minority had appropriate staging. Overall survival of ROH patients at one year is 87.8% compared to 72.5% of patients not seen at the ROH, and was slightly worse at five years with 55.8% survival for ROH patients and 50.6% survival elsewhere. Patients treated at DGHs had significantly more local recurrences.
*Conclusion:* Soft tissue sarcomas are rare. Centralization of treatment improves patients care and improves local control.

**C97 Single Dose Versus Fractionated Irradiation in Metastatic Femoral Bone Lesions: Assessment of Fracture Risks**

Y.M. van der Linden, E.M. Noordijk, P.D.S. Dijkstra, H.M. Kroon
*Leiden University Medical Center, Leiden, The Netherlands*

*Objective:* In randomised Dutch Bone Metastasis Study on palliative effect of single versus multiple fraction irradiation on painful bone metastases all impending fractures were excluded from participation. During follow-up 35 fractures occurred in 1157 patients. 24 fractures developed after single dose 8Gy (4%), 11 after 6 fractions of 4Gy (2%) ($p<0.05$). Whether this difference was caused by insufficient total dose or by lesion-related factors (size, cortical involvement, localisation) was unknown. A subset of

102 patients with femoral lesions, in which 10 fractures occurred in 46 single dose (22%) and 3 in 57 multiple dose patients (5%), was used to assess the pre-treatment fracture risk.

*Methods:* Pre-treatment radiographs were collected. A radiologist, orthopaedic surgeon and radiation oncologist independently and without knowledge of the outcome after treatment (fracture yes/no) scored size and extent, radiographic appearance/structure/aspect, localisation, percentage of cortical involvement (<50% or >50%) and expected risk of fracturing (<50% (low risk) or > 50% (high risk)).

*Results:* In multivariate analysis only estimated risk of fracturing was significant in observers 1 and 2 ($p<.001$ and $p=.001$). Multiple fractions proportionally reduced the risk of fracturing in high risk and low risk lesions in all observers (not significant).

*Conclusion:* Multiple fractions reduce the risk of fracturing, especially in high risk lesions (expected risk of fracturing > 50%) although not significant. Accurate detection of high risk lesions is observer-related. Developing of scoring system to assess risk on fracturing is ongoing.

